# THE INTESTINAL MICROBIOME IN PATIENTS UNDERGOING BARIATRIC SURGERY: A SYSTEMATIC REVIEW

**DOI:** 10.1590/0102-672020220002e1707

**Published:** 2022-12-19

**Authors:** João Kleber Almeida Gentile, Karen Danielle Oliveira, Júlia Guimarães Pereira, Daniel Yuji Tanaka, Giovanna Nagatsuka Guidini, Melissa Zanetti Cadona, Diego Werneck Siriani-Ribeiro, Mariana Tafner Perondini

**Affiliations:** 1 Faculty of Medicine, City of São Paulo University, Surgical Skills and Operative Technique Unit – São Paulo (SP), Brazil;; 2 São Camilo Hospital, Pompéia Unit, Department of Digestive, Bariatric and Metabolic Surgery, São Paulo (SP), Brazil;; 3 São Camilo Hospital, Pompéia Unit, Clinical Nutrition Department, São Paulo (SP), Brazil;; 4 Faculty of Medicine, City of São Paulo University, São Paulo (SP), Brazil.

**Keywords:** Bariatric Surgery, Dysbiosis, Microbiota, Small intestine, Large Intestine, Stomach, Cirurgia Bariátrica, Disbiose, Microbiota, Intestino Delgado, Intestino Grosso, Estômago

## Abstract

**BACKGROUND:**

Dysbiosis of the gut microbiota is frequently found in cases of obesity and related metabolic diseases, such as type 2 diabetes mellitus. The composition of the microbiota in diabetics is similar to that of obese people, thereby causing increased energy uptake efficiency in the large intestine of obese people, maintenance of a systemic inflammatory state, and increased insulin resistance. Bariatric surgery seems to entail an improvement in gut dysbiosis, leading to an increased diversity of the gut microbiota.

**AIMS:**

This study aimed to present a literature review on obesity-associated gut dysbiosis and its status post-bariatric surgery.

**METHODS:**

A systematic review of primary studies was conducted in PubMed, SciELO, BIREME, LILACS, Embase, ScienceDirect, and Scopus databases using DeCS (Health Science Descriptors) with the terms “obesity,” “intestinal dysbiosis,” “bariatric surgery,” and “microbiota.”

**RESULTS:**

We analyzed 28 articles that had clinical studies or literature reviews as their main characteristics, of which 82% (n=23) corresponded to retrospective studies. The sample size of the studies ranged from 9 to 257 participants and/or fecal samples. The epidemiological profile showed a higher prevalence of obesity in females, ranging from 24.4 to 35.1%, with a mean age of around 25–40 years. There was a variation regarding the type of bariatric surgery, migrating between the Roux-en-Y bypass, adjustable gastric banding, and vertical gastrectomy. Of the 28 studies, 6 of them evaluated the gut microbiota of obese patients undergoing bariatric surgery and their relationship with type 2 diabetes mellitus/glucose metabolism/insulin resistance.

**CONCLUSIONS:**

The intestinal microbiota is an important influencer in the regulation of the digestive tract, and obese individuals with comorbidities (diabetes mellitus, hypercholesterolemia, and metabolic syndrome) present important alterations, with an unbalance normal state, generating dysbiosis and the proliferation of bacterial species that favor the appearance of new diseases. Patients who undergo bariatric surgery present an improvement in the intestinal microbiota imbalance as well as reversibility of their comorbidities, increasing their life expectancy.

## RESUMO

**RACIONAL:**

A disbiose da microbiota intestinal é encontrada frequentemente em casos de obesidade e doenças metabólicas relacionadas, como a diabetes mellitus tipo 2. A composição da microbiota em diabéticos é semelhante à de obesos, causando um aumento da eficiência de captação de energia no intestino grosso de obesos, manutenção de um estado inflamatório sistêmico e maior resistência à insulina. A cirurgia bariátrica parece acarretar em uma melhora da disbiose intestinal, levando ao aumento de diversidade da microbiota intestinal.

**OBJETIVOS:**

Este artigo apresenta uma revisão de literatura sobre a disbiose intestinal associada a obesidade e seu status pós cirurgia bariátrica.

**MÉTODOS:**

Uma revisão sistemática de estudos primários foi realizada em bases de dados PubMed, SciELO, BIREME, LILACS, Embase, ScienceDirect e Scopus utilizando o Descritores em Ciência da Saúde (DeCS) com os termos: “obesidade”, “disbiose intestinal”, “cirurgia bariátrica” e “microbiota”.

**RESULTADOS:**

Foram analisados 28 artigos que tinham como característica principal serem estudos clínicos ou revisões de literatura, dos quais 82%, n=23, correspondem a estudos retrospectivos. O tamanho das amostras dos estudos variou de 9 a 257 participantes e/ou amostras fecais. O perfil epidemiológico mostrou haver maior prevalência de obesidade no sexo feminino, variação de 24,4 a 35,1%, com idade média em torno dos 25 a 40 anos. Houve uma variação com relação ao tipo de cirurgia bariátrica, migrando entre a Bypass em Y-de-Roux, Banda gástrica ajustável e gastrectomia vertical. Dos 28 estudos, 6 deles avaliaram a microbiota intestinal de obesos submetidos à cirurgia bariátrica e sua relação com diabetes mellitus tipo 2/metabolismo da glicose/resistência insulínica.

**CONCLUSÕES:**

A microbiota intestinal é um importante influenciador na regulação do aparelho digestivo, e que indivíduos obesos com comorbidades (diabetes mellitus, hipercolesterolemia e síndrome metabólica) apresentam alterações importantes, com desequilíbrio do seu estado normal, gerando disbiose e a proliferação de espécies bacterianas que favorecem o aparecimento de novas doenças. Pacientes submetidos a cirurgia bariátrica apresentam melhora do desequilíbrio da microbiota intestinal, bem como uma reversibilidade de suas comorbidades, elevando a expectativa de vida.

## INTRODUCTION

The human intestinal microbiota (IM) is formed basally from birth to 2 years of age due to endogenous and exogenous factors, such as environmental exposure, diet, contact with other individuals, pathogens, and the use of medications. Thus, the gut harbors diverse microbial communities, with the phyla *Bacteroidetes* and *Firmicutes* prevailing, although viruses, bacteriophages, yeasts, and fungi establish a symbiosis with the host, such as the genera *Clostridium*, *Lactobacillus, Escherichia*, and *Bifidobacterium*. Such communities possess a broad functional spectrum of biochemical activities, and this wealth of microbial genes is important for maintaining metabolic processes and other physiological regulations^14,16^.

In general, the IM remains stable throughout life. However, the microbiome, understood as the microbiota, its metagenome, and the surrounding abiotic environmental conditions can undergo modification processes. Dysbiosis of the intestinal microbiome, that is, an alteration in the balance of the microbial community composition, is associated with obesity, diabetes, and postsurgical adaptations, among other factors^14^.

Within this context, dysbiosis of the gut microbiota is frequently found in cases of obesity and related metabolic diseases, such as type 2 diabetes mellitus (DM2). Currently, studies suggest that a specific microbiota profile may induce obesity in normal individuals and that already installed obesity may contribute to shaping the profile of the existing microbiota. Studies also point out that obesity is related to both lower microbiota diversity and lower microbial gene richness (MGR), with a possible decrease in the *Firmicutes-Bacteroidetes* ratio. The composition of the microbiota in diabetics is similar to that in obese people. It is also attributed to this phenotype of the IM the increased efficiency of energy uptake in the large intestine of obese people, maintenance of a systemic inflammatory state, and greater resistance to insulin^1,13^.

Currently, there has been a considerable increase in the performance of bariatric surgeries worldwide as a way to treat obesity and consequently to reduce cardiovascular risks and diabetes, making this procedure “metabolic surgery.” Conventional bariatric procedures include vertical sleeve gastrectomy (SG), Roux-en-Y bypass (RYGB), and biliopancreatic diversion/duodenal switch (BPD). RYGB and BPD are restrictive and disabsorptive procedures, restricting food intake and reducing intestinal absorption. The SG, on the contrary, is purely restrictive. Bariatric surgery is mainly aimed at long-lasting weight loss, but it is also found in the literature to lead to decreased mortality and improvement of associated comorbidities, such as DM2. Another effect of bariatric surgery, pointed out by studies, is the possible improvement of dysbiosis found in obese patients, which may lead to an increase in the diversity of the IM and the richness of microbial genes, in addition to an increase in specific genera of bacteria. Such changes lead to weight loss, reduced systemic inflammatory status, increased insulin sensitivity, and various metabolic improvements^17^.

The aim of this article was, by employing a systematic review, to analyze the possible influence of bariatric surgery on the composition of the gut microbiota and the metabolic modifications arising from it in postbariatric patients.

## METHODS

This article is a systematic literature review with a qualitative approach that seeks to analyze the changes in the IM of patients undergoing bariatric surgery. In addition, this article is a basic and comprehensive research of data collection from original studies or literature reviews, presentation of results, and confrontation with the current literature.

**Figure 1:**
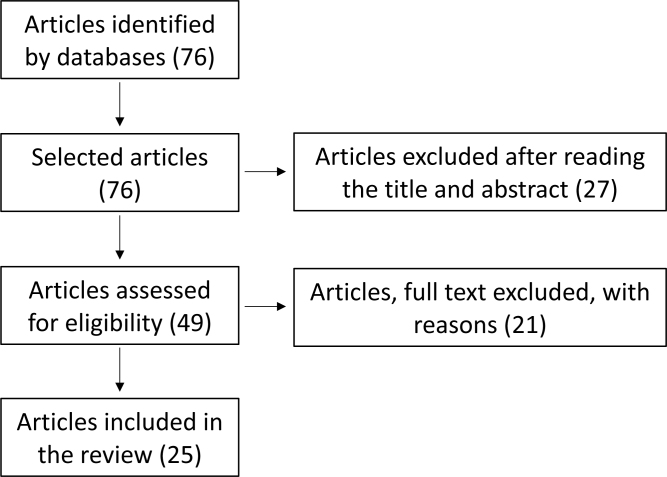
Flowchart of the included articles.

First, a search for descriptors related to the theme was performed, which were identified using the DeCS (Health Science Descriptors). Thus, the following terms were used for the search: “obesity,” “intestinal dysbiosis,” “bariatric surgery,” and “microbiota,” which enabled the formulation of the search, with AND or OR, considering the use of the terms in the title or abstract of the papers. Then, a search was made in PubMed, Google Scholar, and SciELO, and a total of 76 articles were found (Figure 1). After searching for the articles that met the preestablished criteria, the first step was to analyze the title and abstract of the articles, which was performed by two researchers, and then only the articles that were agreed upon by both were selected.

The inclusion criteria were as follows: articles in English or Portuguese that aimed at the theme of the review, that is, the analysis of intestinal dysbiosis in postbariatric patients and recent articles from 2009 to 2022. Furthermore, we used articles with unavailable reading as exclusion criteria, as well as articles that did not meet the research objective, with the main focus on other aspects, such as a simple citation of the postbariatric IM, types of bariatric surgeries, and other related ones that did not assess the change in microbiota after the reduction surgery.

Finally, 28 articles were chosen, which were studied and analyzed, with the aim of explaining the outcome of the theme addressed. The research does not require approval from the Institution's Research Ethics Committee.

## RESULTS

We analyzed 28 articles dealing with clinical studies or literature reviews as their main characteristics, 82% (n=23) of which were related to retrospective studies. The sample size of the studies ranged from 9 to 257 participants and/or fecal samples. The epidemiological profile showed a higher prevalence of obesity in females, ranging from 24.4 to 35.1%, with a mean age of around 25–40 years.

All selected articles exposed the relationship between the IM in obesity and its alteration after bariatric surgery. There was a variation regarding the type of bariatric surgery, migrating between the RYGB, adjustable gastric banding, and vertical gastrectomy (VG). Of the 28 studies, 6 of them evaluated the gut microbiota of obese people undergoing bariatric surgery and their relationship with DM2/glucose metabolism/insulin resistance.

Five studies demonstrated the importance of fecal analysis (metabolome) and genetics, along with epigenetics, for future contributions to improving the gut microbiota in obesity, such as fecal transplantation. One of these articles also brought up the relationship between respiratory diseases, such as asthma, and advanced overweight. Another study evaluated the importance of probiotics in the microbiota after surgical intervention, as well as its association with taste and repercussion on the vaginal microbiota. One of the articles raised the relationship between the IM with cognitive function and the change after bariatric surgery^1,25^.

Finally, we had an article that evaluated the correlation between the IM and bile acids in nonalcoholic fatty liver disease (NAFLD) and its reversal after bariatric surgery.

## DISCUSSION

### Gut microbiome and function

The digestive tract constitutes the largest surface area of the human body, with a size of approximately 30–40 m^2^, and it is uniquely exposed to environmental factors such as diet, antibiotics, pathogens, and other lifestyle habits such as physical activity^1^. The human gut harbors approximately 100 trillion microbes, which have diverse physiological and biochemical functions in the human body^14,16^. The IM is different from one person to another, because its colonization begins at birth and is composed of three phases: from birth to weaning, from weaning to adulthood, and old age. Breast milk is free of bacteria, but soon after birth, the intestine starts to be populated due to endogenous and exogenous influences. During the first 12–24 h, the main bacteria are facultative anaerobes such as *Escherichia coli*, *Enterococcus*, and *Streptococcus*, which will support their growth. A crucial determinant of the development of IM is infant feeding, since at the end of exclusive breastfeeding and the introduction of solid food, there is a greater differentiation of microorganisms, which will result in the adult microbiota. This microbiota remains stable if there are no changes in eating habits, disease onset, or antibiotic consumption. *Bifidobacterium* and *Clostridium* bacteria are more present in children and adolescents compared to adults, while in the elderly, their representation decreases^1,13^.

The IM works as an organ of the human body, whose various functions can suffer alterations if not nourished correctly^14,16,17^. Its main activities are related to the immune system, helping in the intestinal barrier, metabolism, nutrient absorption, vitamin synthesis, defense against pathogens, and the dehydroxylation of bile acid. When there is an inadequate intake of modern ultra-processed Western diets, these functions are affected, diminishing the applicability of the gut microbiome. In contrast, natural and fiber-rich foods assist its development in a healthy and diverse manner^16,17,20,23–25^.

### Profile of the intestinal microbiota in obese and nonobese individuals

The IM is composed of five different phyla that mainly colonize the large intestine, with about 90% of the bacterial species belonging to the phyla *Firmicutes* (*Bacillus* spp*.*) and *Bacteroidetes* (*Bacteroides* spp.). There is also a representation of the other phyla such as *Actinobacteria* (*Bifidobacterium* spp.), *Proteobacteria* (*Escherichia, Helicobacter*), and *Verrucomicrobia* (*Akkermansia* spp.)^1,14,16,25^.

It is known that the profile of IM in obese and healthy individuals is divergent. Obese individuals often have the microbiota associated with a decrease in *Bacteroidetes* and an increase in *Firmicutes*, but some studies in humans have found an opposite ratio, suggesting that the *Firmicutes-Bacteroidetes* ratio would be increased. Low MGR in obese people is associated with metabolic diseases, inflammation, and insulin resistance^16,17^. IM may contribute to obesity in some ways. Its microorganisms participate in the regulation and ability to process nondigestible polysaccharides from the diet, influencing intestinal absorption of short-chain fatty acids (SCFAs). Similarly, there is a regulatory dysfunction of the carbohydrate metabolic pathways of fructose and mannose, galactose, starch, and sucrose. Another particularity would be the gene regulation pathway where, according to the bacterial species present there, there is a promotion of fat storage in adipose tissue (AT)^12,15^. In summary, in obesity, there may be an increase or decrease in bacterial phyla, leading to a change in the relationship between *Firmicutes* and *Bacteroidetes*, causing their diversity to be lower in contrast to individuals within the ideal weight.

According to Abenavoli et al., all carbohydrate and starch metabolic pathways were highly enriched in the obese microbiome. Furthermore, within this observation, the abundance of genes related to lipopolysaccharide (LPS) biosynthesis and peptidoglycan biosynthesis was higher, and this finding could be related to higher levels of inflammatory cytokines such as IL-6 and TNF-beta present in obesity^1^. Finally, pathways related to amino acid metabolism involving phenylalanine, tyrosine, and tryptophan biosynthesis and modules of the glutamine/glutamate transport system were higher compared to those in the control group of healthy individuals^15^.

Butyrate-producing bacteria are in greater numbers in obese individuals, in contrast to the amino acid glycine, which is decreased. Elevated glycine levels are related to improved HbA1c, and acetyl-glycine has been associated with a reduced risk of developing DM2. The bacterial species *Bacteroides thetaiotaomicron* influences host adiposity and metabolism, so its depletion is associated with overweight and serum amino acid concentration^4^. When comparing healthy and obese individuals, one can note a difference in the amount of metabolites derived from gut microorganisms, such as higher production of aromatic amino acids (AAA) and branched-chain amino acids (BCAA). In the microbiota of obese individuals, the serum concentration of phenylalanine, tyrosine, leucine, isoleucine, and valine was notably higher^15^.

In summary, microbial by-products (SCFAs) are produced in the gut and cross the intestinal barrier, proceeding through the blood circulation until they reach the brain. These by-products manage to cross the blood-brain barrier until they reach the hypothalamus, the regulatory center of appetite and metabolic processes. Therefore, the *Enterococcus* species, for example, when fermenting dietary fiber, produce certain SCFAs that are directly related to the decrease in appetite. Thus, direct communication is established between gut and brain^1,3,4,12,15–17^.

### Microbiota, dysbiosis, and relationships with obesity

Obesity is a low-grade but chronic systemic inflammatory disease with AT damage. Inflammation participates directly in the development of complications, since the size of adipocytes influences the production of inflammatory cytokines and chemokines, which recruit pro-inflammatory cells within the AT. Thus, in severe obesity, a high prevalence (75% of patients) of reduced fecal microbiota and MGR occurs^6,7^.

A critical factor in modulating the MGR, and one that can provide diversity, is the eating habits of the individual. In obesity, the intestine is characterized by greater permeability, facilitating the bacterial components to cross the intestinal barrier and fall into the bloodstream, and this would be associated with intestinal dysbiosis, permeability, inflammation, and obesity. Thus, studies show that intestinal dysbiosis is identified in overweight and moderate obesity, which worsens more with increasing body mass index (BMI); the worsening of the disease is associated with metabolic changes, such as insulin resistance, low-grade inflammation, and hypertrophy of adipocytes^6–8,12^.

Abenavoli et al. performed an analysis with mice in which two mechanisms by which the microbiota may contribute to obesity are demonstrated:

1. energy regulation and the ability to process nondigestible dietary polysaccharides, leading to increased intestinal absorption of SCFAs and
2. via gene regulation, promoting increased fat storage in AT.

From this, it is suggested that the microbiota of obese individuals has a greater advantage in the extraction of energy from food when compared to nonobese individuals^1^. Furthermore, analysis of the composition and complexity of the gut microbiota is important to associate IM signatures with host diseases. The two main approaches are metagenomic analyses of random DNA fragment sequencing (Shotgun) and 16S ribosomal RNA gene amplicon sequencing^16^.

Furthermore, through analysis of the fecal microbiome of 1,126 twin pairs, a close relationship between the microbiota and heritable microbial taxa was observed, where the IM of identical twins was more closely related than that of fraternal twins and was observed in other genetically close relatives. The microorganisms themselves contribute to shaping composition through the secretion of peptides and regulatory molecules that influence the metabolic profile of the host. To analyze the effects of genetics and test the relationship of microbial interference with metabolic status, the microbiota of patients with Crohn's disease (CD) was studied in relatives (parents, twins, and non-twin siblings) using DNA fingerprinting. Dysbiosis was present in twin patients with CD and absent in relatives without the disease, even though their genetic heritage was shared. As such, it was found that intestinal colonization by the microbiota results in transcriptional changes in intestinal cells^20^.

### Bariatric surgery techniques and outcomes with gut microbiota modulation

Conventional bariatric procedures include VG or gastric sleeve, gastric RYGB, and BPD. SG involves removing approximately 75–80% of the stomach, leaving a small tubular-shaped pouch. RYGB involves creating a small stomach pouch, usually about 30 ml in volume, and a gastrojejunostomy between the pouch and the jejunum. The ingested food bypasses approximately 95% of the stomach, the entire duodenum, and part of the jejunum. BPD is a disabsortive procedure involving a subtotal gastrectomy plus a long intestinal detour with mixing of bile and nutrients^2^.

Bariatric surgery is substantially recommended by weight loss experts for individuals with BMI 40 or 35 kg/m^2^ with associated comorbidities. All bariatric surgery procedures result in a drastic reduction in food intake and calories consumed^15^. In addition to the anatomical alteration, many patients have preexisting factors that influence postsurgery outcomes, such as gut microbiota, surgical conversion, and AT. With altered gastric physiology, the speed of food transit from the stomach to the ileum changes, and both RYGB and VG decrease the effects of gastric acid on ingested food^2,16^. These changes are disparate according to the surgical technique chosen. Usually, the greatest weight loss occurs after RYGB, with a percentage of approximately 62.58% at 5 years and 63.52% at 10 years^7,16^.

The first study of the microbiota after bariatric surgery occurred in 2009, which included data from three obese individuals, three after gastric bypass, and three with normal weight, and noted an abundance of H_2_-producing bacteria groups. In the same year, another study was done together with the first longitudinal analysis of the gut microbiota of 30 obese individuals undergoing gastric bypass. The results showed that there was a lower *Bacteroidetes/Prevotella* ratio before surgery and a higher *Bacteroidetes/Prevotella* ratio after surgery^17^.

In the literature review, Debédat et al., in 2019, demonstrated that the intestine is a highly plastic and specialized organ for a large surgical removal of the ileum that leads to increased proliferation of cells, thus increasing glucose uptake and utilization^7^. The secretory cells also increase, leading to increased production of intestinal hormones such as glucagon-derived peptide (GLP-2). Postprandial GLP-1 secretion is increased, which may contribute to improved insulin secretion. GLP-2, co-expressed with GLP-1 in intestinal L cells and released after nutrient intake, has a beneficial trophic role in the small intestine with stimulation of crypt cell proliferation, increased intestinal weight, and villus growth in both jejunum and small intestine^2,6–8,16^.

When exploring metabolomic pathways linked to metabolites that could be influenced by bariatric surgery, the surgical intervention reduced tricarboxylic acid cycle, glycine, serine and threonine metabolism, glyoxylate and dicarboxylate, and tyrosine metabolism. These results suggest that BCAAs and aromatics, as well as energy metabolism, were negatively regulated with bariatric surgery^18^. Although beneficial effects of BCAA supplementation have been described, some clinical trials show increased BCAA levels in obesity and in people with insulin resistance^8,11,21^. The RYGB technique induces a reduction in serum BCAAs, further validating how bariatric surgery aids in insulin resistance and glucose metabolism. BCAA levels decreased significantly only in patients who lost at least 10 kg of body weight after bariatric surgery, but not in patients with similar weight loss induced by a restrictive diet, suggesting the role of a bariatric surgery-dependent mechanism in BCAA reduction^6^. The VG technique in particular demonstrated an increased capacity for amino acid biosynthesis after surgery, a mechanism that could be linked to improved glucose control^6,11^.

Some patients undergoing bariatric surgery have reported improvements in metabolic syndrome parameters, including reduced waist circumference, reduced triglycerides, reduced fasting glucose, increased *high-density lipoprotein*, and reduced blood pressure. Many of the explanations for this change in gut architecture are due to altered food intake, hormonal modification, bile acids (BAs), and changes in inflammation levels^5,8,17^. Some of these have been associated with metabolic syndrome parameters and increased *Veillonella*, which is inversely correlated with waist circumference and positively correlated with the percentage of weight loss. *Escherichia*, *Akkermansia*, *Enterococcus*, and *Carnobacterium* positively correlated with percent weight loss and *Bifidobacterium* and *Sutterella* negatively correlated^5,20,26^.

Recently, a meta-analysis by Guo et al. reported the change in two genera, *Escherichia* and *Akkermansia*, after bariatric surgery. Changes in bacterial genera, such as *Eubacterium* spp., *Ruminococcaceae* spp., and *Faecalibacterium* spp., are associated with improvement in metabolic factors, including HbA1c. In addition, the recommended healthy diet after bariatric surgery may also play a role in increasing MGR, in contrast to obesity, whose microbiota richness is reduced^10,16^.

Fusobacteriaceae, Clostridiaceae, and Enterobacteriaceae species increased significantly, while Bifidobacteriaceae and Peptostreptococcaceae decreased in abundance after RYGB. At the same time, there was an increase in microbiota richness, mainly due to *Proteobacteria*, with an increased association between the gut microbiota and white AT gene expression. *Firmicutes* phyla were associated with improved trunk fat mass and glycated hemoglobin^6,19^. In addition, the RYGB showed an increase in the abundance of typically oral tract bacteria such as *Fusobacteria*, *Veillonella*, and *Granulicatella*, and these changes may be substantiated due to pH changes, as when it is elevated in the distal gut, this can affect the gut microbiota. The food flow was also modified, with less nutritive substance in the distal intestine under post-RYGB conditions. In this regard, oxygen plays an important role, as the shortening of the intestinal length in RYGB can lead to the growth of facultative anaerobes such as *Gammaproteobacteria* and a reduction of some obligate aerobes^21^. However, the VG technique did not show significant changes, and the changes in the microbiota induced by surgery occur more gradually and do not completely remodel the microbial genera present in the gut. Finally, when comparing the two techniques, the RYGB has been shown to modify the gut microbiome significantly^19^.

Bypass surgery increases intestinal gluconeogenesis and improves insulin resistance; the increased GLUT1 transporter on the membrane of enterocytes improves glucose uptake, and this occurs mainly to support the increased tissue. The hyperplasia of intestinal cells also explains the increased amounts of glucagon-derived peptides that assist glucose utilization. The sodium-glucose co-transporter (SGLT1) also assists in lowering plasma glucose; this is due to the sodium present in the bile. With this, gastrectomy delayed glucose absorption and did not show intestinal hyperplasia^8^.

However, there is a controversy in the results about this bacterial proportion, as some studies with obese patients show changes in the amounts of microorganisms after surgery, while others show the opposite effect. The bacterial clusters at a few months after surgery changed progressively, and at 3 months, it was possible to observe that the bacteria that made up the microbiome were no longer the same. The changes were not sustained in the long term, returning to the initial preoperative values after 12 months, but the improvement in metabolism persisted. Thus, at 12 months after surgery, the bacterial profile was similar to that of the preoperative one^5,19^. Other postoperative subjects did not have this rapid change in the microbiome after surgery, but improvement in metabolism was achieved after 1 year. These differences show that microbiome change alone is not able to sustain the benefits achieved in the long term, as bacterial groups act synergistically to enhance or degrade metabolism^21^.

Shao et al. conducted a research with rats that underwent the bariatric surgery procedure of VG type and RYGB. After a postprocedural observation for 1, 3, 6, and 9 weeks, they found the effectiveness of bariatric surgeries was associated with their effects on the gut microbiota, as there was a change in the composition, gene content, and fermentation profiles of microbes in the gut, promoting decreased overall adiposity, rapid improvement in glucose metabolism, and remission of obesity comorbidities after the procedure^21^.

### Biomarker discovery

The phenotype of circulating microorganisms and biomarkers occurs disparately and throughout the body. To investigate the existence of biomarkers that are associated with different outcomes in each surgical technique, it was found that the bacterial genus *Veillonella* might be the most characteristic biomarker of the RYGB procedure, while *Blautia* might be the biomarker of VG surgery. Evaluating the postoperative microbiota profile, the RYGB technique was shown to alter the microbiome more abundantly. Thus, the signal transduction pathway was benefited as well as the degradation and metabolism of xenobiotics. Other pathways altered after surgery were decreased amino sugar and nucleotide sugar metabolism, alanine, aspartate and glutamate metabolism, and glycolysis/glyconeogenesis^19,20^.

There is a direct influence between certain bacterial species and surgical techniques. It was found that in RYGB, in addition to weight loss, changes in the profile of the microbiota were made possible. *Firmicutes* phyla were associated with better levels of glycated hemoglobin and improved AT in the trunk. In contrast, the VG technique demonstrated a link to *Akkermansia muciniphila* species, which are related to improved metabolism, decreased adiposity, and inflammation. *A. muciniphila* produces metabolites derived from its fermentation that serve as a substrate for other bacteria^19^. The findings evidenced by the study from the Autonomous University of Barcelona and Chicago teaching hospitals showed that changing the microbiome alters metabolism as a whole, bacteria alter circulating biomarkers, and the relationship between gut microbiome and improved whole-body metabolism. The study looked at 26 patients, 8 of them with DM2, 9 taking metformin, 11 treated for hypertension, 10 for dyslipidemia, and only one had a history of cardiovascular disease. The profile of the microorganisms was improved, but this change could not be sustained in the long term. BAs and butyrate had their bacterial composition progressively altered as the months passed postsurgery^20^.

Metabolites derived from bacterial metabolisms such as BAs, SCFAs, trimethylamine *N*-oxide (TMAO), betaine, and choline have had their activity altered, resulting in benefits to patients; high amounts of TMAO, for example, are associated with a high risk of cardiovascular diseases^21^. Regarding BCAA, there is evidence that supplementation has beneficial effects; however, these levels of obesity, insulin resistance, and DM2 are increased. BCAA-mediated insulin resistance occurs because it inhibits the phosphorylation of the insulin receptor (IRS-1). Patients with insulin resistance not only have increased levels of BCAA but also have in their microbiota good amounts of microorganisms of the species *Prevotella copri* and *Bacteroides vulgatus*, which are species related to the increased expression of genes that perform the biosynthesis of BCAA^3^.

BAs are synthesized in the liver, stored in the gallbladder, and secreted into the duodenum in response to nutrients and their absorption requirements. They act as surfactants and are important in the breakdown and absorption of substances and stimulate the secretion of the hormone GLP-1, synthesized by the L cells of the intestine. They are also natural ligands of the TGR5 receptor, expressed in L cells, which are related to improved glucose utilization, consequent weight loss, and decreased inflammation^6,8^. BAs are able to regulate the composition of the gut microbiota. Therefore, they play an essential role in maintaining a healthy gut microbiota, insulin sensitivity, innate immunity, and balanced lipid and carbohydrate metabolism. The erroneous bioconversion of primary BAs (PBA) into secondary BAs (SBA) results in fecal dysbiosis as well as a metabolic dysfunction and is mechanistically associated with gastrointestinal carcinogenesis, including colorectal cancer and hepatocellular carcinoma^17^.

The mechanism of BA in the face of bariatric surgery-induced improvements in body weight and metabolism includes reduced systemic and hepatic inflammation, which has, as a consequence, more efficient insulin signaling without increased GLP-1 and insulin secretion^6^. The relationship between BA metabolism and gut microbiota is in the *Farnesoid* X receptor (FXR). Patients undergoing gastric bypass developed a “healthy” gut microbiota, with decreased *Bacteroides* and increased *Roseburia*. Such changes were not found in FXR knockout mice and suggest an FXR-dependent signaling pathway. Another finding is that the microbiota metabolizes BAs, which are responsible for the formation of PBA and SBA that activate FXR, thus stimulating the release of gut-derived hormones such as fibroblast growth factor-19 (FGF19), which in turn stimulates BA in its synthesis, increasing host energy consumption. Therefore, elevated levels of FGF19 and BAs are found in adults undergoing gastric bypass surgery after 1 month. The same does not occur in obese adults who have received medication^17^.

Patients with NAFLD have high levels of BA in plasma, as do bariatric patients, but the PBA/ABS ratio in bariatric patients tends to be lower compared to that in NAFLD patients. Apparently, this finding is unrelated to postoperative weight loss, since BA levels decrease after weight loss with caloric restriction and with adjustable gastric banding^21,22^. The intestinal anatomical alteration in the RYGB technique causes poorly digested food to reach distal parts of the small intestine with a higher concentration of BA. A similar effect is found in the SG technique. An intestinal adaptation occurs so that ileal reabsorption of BA is more efficient, justifying the increase in plasma value after bariatric surgery^11,26^. In other words, even if most of the experimental studies performed on this topic are with animals and observational studies are limited, it is known that bariatric surgery plays an important role in the etiopathogenesis of NAFLD. Therefore, the modulation of the gut microbiota after bariatric surgery and the influence on the PBA/SBA ratio in plasma induce metabolic improvements that do not necessarily depend on weight^2,24^.

As described earlier, the microbiota has a great influence on the immune system of the human body. Obesity is a disease of low-grade inflammatory character that generates lesions in the AT, which can result in moderate and chronic systemic inflammation. They influence the production of inflammatory cytokines and chemokines that recruit inflammatory cells within the AT^6,8^. This increase in inflammatory processes can weaken the gut wall^7^, causing many obese patients to have dysbiosis of the gut microbiota^14^. Macrophages are the main inflammatory cells in the AT and exhibit a mixed surface marker in obese people. The continuous inflammatory cycle and abnormal metabolism are induced by the action of lymphocytes, mast cells, and neutrophils^14^. Some chemokines such as MCP1 also assist in the recruitment of pro-inflammatory cells^23^. The expression of pro-inflammatory cytokines such as IL-1α, IL-6, IL-8, MIP-1α, IFN-α, and TNF-α are significantly increased in obese women^10,26^.

There was a reduction in adipocyte size, after bariatric surgery, accompanied by a reduction in the number of pro-inflammatory cytokines such as CD4, CD16+, and monocytes, resulting in a decrease in body weight and metabolic improvement. Changes in the receptors present on monocytes, such as the TLR4 receptor, which is able to recognize bacterial LPS, are associated with modification of the microbiota. Th1 lymphocytes have also changed their phenotype. In obesity, they had an inflammatory profile and a Th1/Th2 ratio that changed after surgery^8^. Consequently, as improvements in blood glucose are associated with higher amounts of Th2, in general, the profile of inflammatory secretion tends to decrease after bariatric surgery^8^.

The glucose levels improve within a few days after gastric bypass, and the caloric restriction after surgery leads to weight loss and also changes in gut hormones that are anorexigenic, such as GLP-1, CCK, PYY, OXN, and eventually glycine. The amount of weight lost also influences the remission of diabetes. Furthermore, bariatric surgery with its consequent weight loss improves insulin sensitivity, specifically when compared to gastric bypass versus a restrictive diet^6,8^. In addition to controlling food intake, gut hormones affect insulin secretion and resistance. GLP-1 is the most studied hormone after bariatric surgery, and patients with DM2 have decreased secretion of this hormone in the postprandial period^18,26^.

The secretion of GLP-1 increases after bariatric surgery, improving the response to insulin. In a literature review, Debédat et al. evidenced that after cases of hypoglycemia caused by an increase in the number of beta cells after bariatric surgery, the pancreas performed an adaptation after surgery, thus regulating possible complications^8^. The mass of beta cells had changed in the pancreas of patients who underwent gastric bypass, and they suffered from neuroglycopenic symptoms of postprandial hypoglycemia. However, the findings are heterogeneous; another study on obese patients found that there was an increase in pancreatic beta-cell mass before and after performing VG and gastric bypass on a total of 26 patients with severe obesity, some with DM2 and others without diabetes. Despite the improvement in glucose homeostasis, even with surgery, the beta-cell function is not recovered, and it does not start to act as in people who have always been thin, even with the weight loss of the bariatric patient^2^.

GLP-2 also seems to assist in metabolic improvements, and its amount increases significantly after gastric bypass. This hormone has a protective effect on the intestinal barrier and inflammation^8^. Another gut hormone that bariatric surgery interferes with is ghrelin, an orexigenic peptide produced by stomach cells. Plasma levels of ghrelin increase before meals and decrease postprandial. With diet-inducing weight loss, ghrelin levels increase and this would be related to regaining the weight lost after dieting, whereas after gastric bypass, plasma levels of ghrelin have been shown to be lower^2^. Ghrelin is lessened by VG, which also reduces weight gain and food intake and enhanced response to the GLP-1 hormone. PYY hormone is an anorexigenic hormone secreted by cells in the ileum and colon in response to nutrient stimulation; it has an appetite-suppressing effect in obese individuals. Finally, foregut and anti-incretin reduce a pathophysiological increase in anti-incretin signaling that normally serves to prevent postprandial hypoglycemia by neutralizing incretin-mediated insulin^2^.

### Metabolites after bariatric surgery

The gut microbiota has several functions, such as biochemical activities that affect the human body, including metabolite production, physiological regulation, and interaction with the host cellular response and immunity. In addition, the gut is uniquely exposed to changing environmental factors such as dietary habits, antibiotics, pathogens, and other conditions such as physical activity^20^. Microorganisms present throughout the digestive tract assist digestion and ferment complex carbohydrates and amino acids, produce SCFAs, such as acetate, propionate, and butyrate, and contribute to lipid and amino acid metabolism, protein digestion, and energy balance^17,20^.

Among the components metabolized in the gastrointestinal tract (GIT), 30 were modified after bariatric surgery. There was a 50% reduction, mainly related to metabolites of the amino acid pathway, BCAA, phenylalanine, and tryptophan. In the 1-year bariatric period, these metabolites were associated with body composition change^12,15^.

The microbiota allows for energy extraction from indigestible sources such as fermentation of carbohydrates and fiber and production of SCFAs, which is equivalent to 10% of an individual's daily energy requirement^12^. SCFA serves as an energy source for the colon epithelium, liver, and peripheral tissues, and it can be converted by the liver into triglycerides, which can be taken up and stored in adipocytes. However, there is no unanimity regarding the role of SCFA and its influence on obesity. While studies point to high levels of SCFA in obese individuals, others point out that SCFA acts as a regulatory molecule beneficial for metabolism^12^.

The composition of the microbiota helps maintain the integrity of the intestinal barrier; for example, *A. muciniphila* regulates the translocation of microbial molecules through the gut, but this increased bacterial population may be associated with certain pathologies, and more studies are needed to understand the role of the microbiota in maintaining the intestinal barrier^17^. Gastric bypass surgery is associated with decreased plasma levels of LPS, inflammatory markers, and C-reactive protein. After bariatric surgery, metabolites from the microbiota, such as butyrate, modulate the immune system, stimulate intestinal epithelial cells to produce TGF-beta, and activate Treg (anti-inflammatory) cells^17,20^.

A high-fat diet may be involved in the reduction of *Bifidobacterium* spp. and hyperactivation of the endocannabinoid system. These factors may impair the normal microbial composition of the gut and cause increased intestinal permeability. In one of the studies^15^, 148 metabolites, including glutamate, phenylalanine and tyrosine, differed in abundance between cases and controls^6,7^. In canonical correspondence analysis, the metagenomic linkage groups of the case- and control-enriched were clearly separated by those of the vectors for tyrosine, phenylalanine, glutamate, and BCAAs, indicating the association of these amino acids with the altered microbiome in obesity. Substantially, this analysis is consistent with the greater capacity for AAA and BCAA production in the microbiota of obese individuals, and the serum concentration of phenylalanine, tyrosine, isoleucine, leucine, and valine was considerably higher in obese individuals than in lean controls^26^.

The main source of SCFAs is in dietary fiber intake, and according to cumulative epidemiological data, there is a protective effect of high intakes of these fibers for maintaining a healthy body weight. The mechanism of how SCFAs are involved in the development of obesity is related to providing an additional source of energy that contributes to fat deposition in the body and is spent on binding G-protein-coupled receptors (GPCRs), GPR41 and GPR43, that regulate energy^12^. In addition to adipogenesis, they also regulate fasting-induced adipokines. SCFAs regulate glucose homeostasis and leptin secretion via the free fatty acid receptor FFAR3, which modulates satiety. Studies suggest that increased SCFA concentrations in the feces of overweight and obese individuals may be due to increased conversion of amino acids to SCFA and that obesity-related changes in SCFA amounts may reflect increased microbial amino acid catabolism^12^.

### High-fat diets and intestinal permeability

High-fat diets can lead to intestinal dysbiosis, increasing permeability and interference with sensory systems such as taste, as well as contributing to inflammation and metabolic changes. Therefore, the microbiota can provoke behavioral responses in the absence of an immune response^6,14,17^.

After bariatric surgery, there is an increase in LPS synthesis within the IM that is not associated with systemic inflammation, suggesting that there is less translocation of LPS into the systemic circulation through a reduction in intestinal permeability after the procedure^10^. DM2 and obesity normally provide an increase in intestinal permeability due to LPS molecules from Gram-negative bacteria that influence inflammation of the intestine. However, after the surgery, there is an improvement in the intestinal barrier due to the patient's lower fat consumption and an increase in *Akkermansia* bacteria, a microorganism that aids glucose metabolism^8^. Regarding the metabolites produced by the bacteria, an increase in TMAO, alteration in the metabolism of tryptophan, heme, and phenylalanine, and a decrease in LPS (a product that increases intestinal permeability) were noted, with the amount of LPS tends to decrease after 90 days of operation^25^.

Bariatric surgery has been indicated for weight loss in obese patients, and the term “metabolic surgery” has come into use to describe a gastrointestinal surgical intervention to treat DM2, an obesity-related comorbidity^17^. Randomized studies have identified improved glycated hemoglobin levels after surgery, but with different findings according to surgical technique. Patients who underwent bypass were able to remain without glycemic control medication when compared to patients who underwent VG^8^. Relapse in glycemic control after surgery does occur but over a longer period. One year after bariatric surgery, glycemic values are not the same, and 30–50% of patients had a relapse of DM2. The parameters such as age and DM2 severity are some of the factors that may have influenced this relapse, but glycemic values are still better compared to the preoperative metabolic state^8^.

Obesity causes injury to the AT, which can lead to low-grade systemic inflammation. The size of adipocytes is related to inflammatory factors such as cytokine recruitment and inflammatory cells^6^. Macrophages, lymphocytes, mast cells, and neutrophils contribute to a continuous inflammatory cycle and abnormal metabolism in obese patients, but macrophages are the inflammatory cells that are in the greatest numbers within AT^6^. After gastric bypass, glucose levels improve within a few days, caloric restriction leads to weight loss, and there is an alteration of gut hormones, such as GLP-1, CCK, PYY, OXN, and eventually ghrelin, which are anorexigenic^8^.

Changes in pancreatic hormones and beta cells influence the improvement of DM2 in bariatric patients. A weight loss of around 20% of initial body weight showed an improvement in beta-cell function after RYGB, and patients with DM2 exhibit an altered incretin effect due to reduced postprandial GLP-1 secretion. After metabolic surgery, the secretion of this hormone increases, improving the insulin response. However, the beta-cell function has not been recovered with the same functioning in healthy individuals, as it continues to act dysfunctionally even with glucose improvement^14^. Another intestinal hormone, GLP-2, also seems to help in metabolic improvements, as its amount increases significantly after the gastric bypass. This hormone has a protective effect on the intestinal barrier and inflammation, so the bypass technique increased intestinal neoglycogenesis, improving insulin resistance^9^.

Recent evidence shows that after the bypass technique, patients have decreased plasma glucose through SGLT1. This sodium, contained in the bile, assists in the uptake of glucose through the intestine, thus decreasing its amount in the plasma. The increase in intestinal cells explains the greater amounts of glucagon-derived peptides secreted, thus improving glucose utilization^8^. Metformin is one of the main drugs used to treat DM2, as it accumulates in the intestinal mucosa at levels 300 times higher than the blood levels. Metformin has demonstrated its modulatory effect on the microbiota, increased both the abundance and activity of *Akkermansia*, improved the *Bacteroidetes/Firmicutes* ratio, and reduced the inflammation markers IL-6 and IL-1β in AT^20^.

### Modulation of the intestinal microbiota in obesity: postbiotics, prebiotics, probiotics, and fecal transplantation

The prebiotics and probiotics are potential modulators of obesity and comorbidities and may be useful for homeostasis of the microbiota and consequent improvement in obesity and comorbidities^3^. The beneficial effects of probiotics are associated with three mechanisms:

1. stimulation of beneficial bacteria and SCFA production, resulting in improved barrier and resistance to inflammatory stimuli;
2. increased amounts of beneficial species to restore intestinal dysbiosis; and
3. modulation of lipid metabolism, through inhibition of lipogenic enzymes and decreased lipoproteins and triglycerides^3^.

The use of probiotics increases the diversity of the IM, selecting beneficial microorganisms, as well as reducing intestinal permeability and improving inflammation^17,20^. Supplementation with prebiotics and probiotics enriched bacterial growth, vitamin B12 bioavailability, and weight loss after bypass. The use of probiotics reduces gastrointestinal complaints after a bariatric procedure. However, inflammatory markers appear to have been unaffected^19^.

Fecal microbiota transfer (FMT) has been used as a treatment option for obesity and other metabolic diseases^23^. It is an option to modify the IM, improving its diversity; however, it is used only in *Clostridium difficile* infection, resulting in improved clinical response or even cure of this disease in 80–90% of cases with 60% complete remission 1 month after FMT in patients of different age group^20,23^. The technique consists of transferring a fecal suspension from a healthy individual into the GIT of a sick individual; however, there is still a lack of sufficient studies for its use in the treatment of obesity^9^. Such therapy was and still is used in most research studies, using nasojejunal tube, colonoscopy, or enema, sometimes requiring multiple administrations to be efficient in some diseases^23^. Thin donor FMT improves insulin sensitivity in obese people with metabolic syndromes. In the first study that used FMT in the control of DM2, it showed a significant improvement in peripheral insulin sensitivity as assessed by the gold standard, which is an euglycemic-hyperinsulinemic stable isotope. The main reason for using FMT to induce weight loss is because the gut microbiota of obese people captures more energy compared to nonobese individuals with the same caloric intake^17,18,20^.

Microbial colonies locate in strategic regions near taste receptor cells and in the gut to create optimal communication and may influence host-feeding behavior via the gut-microbiota-brain axis. The role of taste receptors in nutrient sensing and the resulting impact on taste and digestive processes suggests that by-products of gut microbiota metabolism, such as SCFAs, may also act as stimulators of taste receptors. Interruptions in the composition of the gut microbiota lead to changes in taste^14^.

Among the many factors involved in appetite and satisfaction, the bacteria in the digestive tract, from the mouth to the distal parts, play a key role in regulating taste responses. Perception can be defined as the strength with which a signal is transduced and can be considered in terms of sensitivity and restriction; the same taste receptors on the tongue are also present in the GIT. These receptors then translate nutrient signals into neuropeptide hormones, vagal activation, or nutrient utilization^13,14^. Regarding taste modulation, the literature indicates two likely pathways, one via the immune system and the other via hormone secretion. The presence of commensal microorganisms near taste and nutrient-sensitive cells can generate an immune response, whereas microorganisms present in the gut microbiota interfere with endocrine function by two routes: directly through bioactive metabolites such as SCFAs and indirectly as a modulator of inflammatory, immune, and hormonal response^14^.

This pattern of receptor abundance determining perception is rooted in observations of the number of taste buds on the papillae and tongue, which predict taste intensity. In extrapolating this to eating behavior, the general assumption is that the lower the sensitivity to taste, that is, hyposensitivity, the greater the taste stimuli that are consumed to obtain the same stimuli. The connection between tastes should also be noted for super tasters. Hypersensitivity to bitterness leads to an aversion to vegetables, while hypersensitivity to sugar leads to increased hedonic responses and greater consumption of sweets. For example, microbial acid production does not create a constant sensation of acidity but increases its thresholds through sensory adaptation; in this context, it has been observed that taste ability in elderly hospitalized patients was reduced, particularly in those who had increased amounts of *lactobacilli*. Other phyla of bacteria are related to increased taste sensitivity; *Actinobacteria* and *Bacteroidetes* are especially related to bitter taste^14^.

While oral microbes must endure a period of time without exogenous nutrients, intestinal microbes have a continuous source of nutrients in the form of dietary fiber. Although taste receptors are abundantly expressed in the oral cavity, they are also present in extra-oral tissues and are involved in many metabolic functions, such as chemosensitization, perception, appetite hormone release, and other gastrointestinal functions. Changes in the intestinal luminal environment cause specialized cells to secrete intestinal peptides, such as CCK, GLP-1, GP-2, and PYY. This process is regulated by cell surface GPCRs, which are expressed in the apical domain of the specialized cells and have a high degree of specificity for nutrients and taste, as well as for microorganisms and toxic compounds^2^.

Popular nutritional evidence states that it is possible to modulate the taste buds by adhering to a diet restricted in sugar, salt, and fat; as in a randomized study, there was a change in the intensity of sweetness perceived by individuals who stayed for a while on a sweet-restricted diet. This modification in the taste signaling mechanism may lead to increased consumption of preferred food substrates by specific microbes for their own survival. Taste plays an important role in food choices and is the most important factor in food choice and consumption^14^.

### Antibiotics and obesity

Antibiotics have the property of modulating the gut microbial communities in the first moment and possibly in the medium and long term, and they may induce gut dysbiosis. Exposure to antibiotics early in life can be seen as a risk factor for obesity^4,17,20^. Diseases such as *C. difficile* infection induced by the use of multiple antibiotic regimen lines are examples of indication for fecal microbiota transplantation, as already discussed^23^.

### Relationship between altered gut microbiota after bariatric surgery and memory

Bariatric surgery is the gold standard in surgical treatment against obesity. This condition is most often related to a condition of gut dysbiosis and bariatric surgery aids in improving this condition as well as cognitive function, including memory decline. Evidence in animals and humans links hypercaloric diets with gut dysbiosis, which in turn affects brain structure and cognitive function^26^.

There are a few studies shedding light on obesity-related memory impairment and reversal with weight loss. Enaud et al. reported that Rey's Auditory Verbal Learning Test (RAVLT) scores, a neuropsychological instrument used in clinical practice to detect memory problems, in dementia and predementia and duodenal switch (DS) significantly improved after bariatric surgery^10^. RAVLT responders had a lower history of DM2 while DS responders tended to have a lower homeostatic model assessment index — this mathematical model predicts the level of insulin resistance and functional capacity of pancreatic B cells according to lower baseline blood glucose and insulinemia. In RAVLT responders, alpha bacterial diversity, Simpson's Index, an index that measures community diversity, was significantly higher after bariatric surgery compared to nonresponders^10^. Significant increases in *Ruminococcus* and *Prevotella* sp. were associated with improved RAVLT scores^26^.

Regarding microbiota, *Agaricus blazei*, *Rhodotorula*, *Bipedia*, *Malassezia*, and *Mucor* were strongly associated with improved RAVLT scores after bariatric surgery. Increases in *Prevotella* and *Parabacteroides* were associated with improvements in DS, while decreases in *Clostridium* and *Akkermansia* were associated with improvements in working memory. Reductions in *Clostridium*, *Akkermansia*, *Bipedia*, and *Candida* sp. were also observed to be associated with improved DS scores^26^.

## CONCLUSION

The IM is an important influencer in the regulation of the digestive system, and obese individuals and/or those with comorbidities (e.g., diabetes mellitus, hypercholesterolemia, and metabolic syndrome) present with altered microbiota, unbalancing its normal state, generating dysbiosis, and proliferation of bacterial species that favor the onset of new diseases. Research shows that changing the microbiome alters the metabolism as a whole, bacteria alter circulating biomarkers, and the relationship between gut microbiome and improved metabolism of the whole body.

Bariatric surgery is a procedure to remodel this microbiome and, regardless of its technique, it does not completely follow the phenotype. However, the present article shows that patients who undergo the surgical procedure show improvement in the imbalance of the gut microbiota as well as reversibility of their comorbidities, thus raising their life expectancy and lifestyle.
